# Microstructural Changes Caused by the Creep Test in ZK60 Alloy Reinforced by SiC_p_ at Intermediate Temperature after KOBO Extrusion and Aging

**DOI:** 10.3390/ma16103885

**Published:** 2023-05-22

**Authors:** Yang-Yang Wang, Chen Jia, Min Xu, Mosab Kaseem, Morteza Tayebi

**Affiliations:** 1 School of Engineering, Xi’an Siyuan University, Xi’an 710038, China; 2 Xi’an Aerospace Propulsion Test Technology Institute, Xi’an 710100, China; 3 Corrosion and Electrochemistry Laboratory, Department of Nanotechnology and Advanced Materials Engineering, Sejong University, Seoul 05006, Republic of Korea; 4 Young Researchers and Elites Club, Science and Research Branch, Islamic Azad University, Tehran 14778-93855, Iran; morteza.tayebi@srbiau.ac.ir

**Keywords:** ZK60/SiC_p_ composite, grain boundary serration, kink band, shear band

## Abstract

In this study, we investigated the creep properties of ZK60 alloy and a ZK60/SiC_p_ composite at 200 °C and 250 °C in the 10–80 MPa stress range after the KOBO extrusion and precipitation hardening process. The true stress exponent was obtained in the range of 1.6–2.3 for both the unreinforced alloy and the composite. The apparent activation energy of the unreinforced alloy was found to be in the range of 80.91–88.09 kJ/mol, and that of the composite was found to be in the range of 47.15–81.60 kJ/mol, and this indicated the grain boundary sliding (GBS) mechanism. An investigation of crept microstructures using an optical microscope and scanning electron microscope (SEM) showed that at 200 °C, the predominant strengthening mechanisms at low stresses were the formation of twin, double twin, and shear bands, and that by increasing the stress, kink bands were activated. At 250 °C, it was found that a slip band was created in the microstructure, and this effectively delayed GBS. The failure surfaces and adjacent regions were examined using SEM, and it was discovered that the primary cause of failure was cavity nucleation around precipitations and reinforcement particles.

## 1. Introduction

Some magnesium alloys have superior mechanical qualities, such as high specific strength at room temperature and superplastic behavior at moderate temperatures, making them suitable choices for use in aviation and automobile engine components [1,2,3,4]. The suitability of Mg is due not only to its relatively low density, which enables a reduction in vehicle weight [5,6], but also to a variety of other features, such as its good damping characteristics, its dimensional stability, and the ease by which it can be machined and cast. These advantages have made it economical to replace alloys such as stainless steel with Mg alloys [7,8]. The high-temperature behavior of materials is assessed using homologous temperatures, and the low melting temperature of Mg alloys (780 °C), which causes creep in Mg engineering applications, is a major failure mechanism. Many factors, such as temperature, grain size, and servicing time, affect the type of creep mechanism and the effective elongation [2,9]. In Mg alloys, due to the presence of weak grain boundaries, the GBS mechanism is more prevalent than other creep mechanisms at low stresses. In polycrystalline materials, grain boundaries act as a barrier to mobile dislocations, and by decreasing the grain size, the strength and yield stress can be increased. This is because more stress is needed to activate dislocations than to activate other deformation mechanisms (such as GBS) [10]. GBS is a process of sub-deformation that occurs at high temperatures and in fine-grained materials, and it is mainly observed in Mg alloys at medium and high temperatures. Studies have shown that with reduced grain size, the likelihood of GBS increases, and this is a major factor leading to the increased elongation of fine-grained alloys [11]. Intergranular sliding is a major factor that leads to GBS activation. GBS, in addition to activating the superplastic mechanism, causes the nucleation of cracks in samples [12].

The stacking fault energy is very low in Mg alloys (about 78 mg/m^2^). On the one hand, this causes refinement through recrystallization, and on the other hand, it causes grain growth at high temperatures. One way to stabilize the microstructure is to create precipitation by adding alloying elements [13,14]. The modification of the microstructure using mechanical means is another solution [15,16]. The aging and extrusion behavior of ZK60 alloy is fully explained in [17]. Recent advances, however, have influenced the compositing of Mg alloys. These composites have abundant potential and provide high performance in aerospace and automotive applications due to their high dimensional stability and high strength. Load transfer is a key mechanism for increasing the creep resistance of magnesium-based composites because it reduces the strengthening effect of a reinforcement by increasing the applied stress. It has been shown that increasing the length to diameter ratio of a reinforcement increases its the load-bearing capacity, and this is why short fibers and whiskers are superior to particles. However, since the price of whiskers and fibers is very high, they are rarely used as structure parts. It is thus important to strengthen the properties of composites reinforced with particles.

There are few studies on the high-temperature creep properties of ZK60 matrix composites. Chmelik et al. [18] studied the creep properties of unreinforced alumina short fibers and AZ91-reinforced alumina short fibers and showed that adding alumina short fibers to AZ91 alloy improves creep resistance. Shamekh et al. [19] examined an AZ91D matrix composite reinforced with TiB_2_-TiC. Their results showed that reinforcement increased the elastic modulus and improved compressive creep behavior. Elongation, on the other hand, reduced. Dieringa et al. [20] examined AE42 magnesium alloys reinforced with 20% Saffil fibers. Their results showed that the creep strength of the composite increased dramatically compared with that of the matrix alloy. The creep properties of the samples produced in the direction of the fibers were better than the properties of the samples produced in the vertical direction. This difference indicates that the orientation of the fibers affects the creep properties. The presence of short-fiber reinforcements led to a two- to three-fold decrease in creep rate in an AZ91/Saffil composite due to the division of the applied load between the matrix and the reinforcement [21].

Although magnesium matrix composites exhibit good strength properties compared with unreinforced alloys, their elongation is not satisfactory. However, magnesium with an HCP crystal structure is also inherently low formability. The elongation of composites can be improved by inducing superplasticity. Limited data are provided on the superplastic behavior of Mg-based composites. Kim et al. [22] reported a 100% improvement in elongation in a 13% SiC AZ91 composite produced by squeeze casting. Nieh et al. [23] observed the superplastic behavior of a ZK60A composite reinforced with 17% SiC particles produced using powder metallurgy. Mukai et al. [3] reported an improvement in traction ductility at room temperature in a 17% SiC ZK60 composite during extrusion, which improved and modified the microstructure through refinement. The aim of this study is to investigate the effect of the superplastic behavior of ZK60 alloy and a ZK60/SiC_p_ composite in order to improve the elongation caused by extrusion during creep at temperatures of 200 °C and 250 °C in the 10–80 MPa stress range.

## 2. Materials and Methods

### 2.1. Composite Preparation

ZK60 alloy with a chemical composition of Mg-5.1Zn-0.48Zr (wt%) as a composite matrix and β-SiC particles (40 μm, purity of more than 99%) at 10 vol% as the reinvestment phase was used. The composite was prepared using the stir casting method in an induction furnace at 760 °C. A protective CO_2_ + SF_6_ atmosphere was used to prevent oxidation. After the alloy was homogenized, SiC particles were added to the melt using the vortex method. Details of the composite preparation method are described in [2,24]. To enable an accurate comparison of the results, the ZK60 alloy was cast under the same conditions as the composite. The casting ingots were homogenized at 400 °C for 8 h. Following homogenization, the samples underwent KOBO extrusion at a starting temperature of 40 °C, a ratio of 1:12, and a speed of 0.1 mm/s, with a 5 Hz die oscillation frequency and a 7° die rotation angle. Details of the KOBO extrusion process are described in [2,5,25]. Precipitation hardening was performed at 175 °C for 12 h., and details of the method used are described in [17,26].

### 2.2. Creep Test

An accelerated creep test was used to conduct the creep test at temperatures of 200 °C and 250 °C in the 10–80 MPa stress range. The SANTAM STM-100 model (Santam Engineering Design Co., Tehran, Iran) was used to carry out the creep test in accordance with the ASTM EB9-83 standard. The sample dimensions (a length of 20 mm and a diameter of 4 mm) were chosen in accordance with ASTM E8. The elongation during the test was calculated using a back-to-back model strain gauge with a linear potentiometer sensor and an accuracy of 1 µm. An extensometer was used to increase the accuracy of the elongation calculation. A furnace with three different temperature zones was used, and during the tests the samples were placed in zone II of the furnace, which had a thermal stability of ±1 °C. In order to bring each sample to the test temperature, they were kept at the desired temperature for 30 min before applying the load. The stepwise loading method was used to determine the minimum creep rate; at the beginning of the test, a low load was applied, and after the steady state region was established, an increase in the load was applied to the sample. This increase continued until the sample fractured. The stress levels applied to the samples during the accelerated creep test at different temperatures until the fracture stage are reported in Table 1. Additionally, in order to characterize the microstructure of the crept sample at a specific temperature and stress, three distinct samples were tested separately at a single stress and a single temperature.

### 2.3. Characterizations

The microstructures of the samples after conventional metallography was analyzed using an Olympus optical microscope (Olympus Corporation, Tokyo, Japan) and a Philips X130 scanning electron microscope (SEMTech Solutions, North Billerica, MA, USA). To investigate the grain size, a solution [17] of 5 mL of acetic acid, 6 g of picric acid, 10 mL of water, and 100 mL of 95% ethanol was used. MountainsSPIP^®^ (Image Metrology) software (version 9, Digital Surf, Besançon, France) was used to analyze the microscopic images.

## 3. Results and Discussion

### 3.1. Microstructure

**Figure 1:**
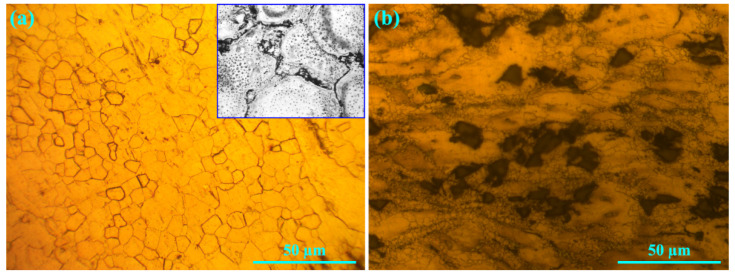
Microstructures of the samples after KOBO extrusion and aging: (**a**) unreinforced alloy and (**b**) composite.

Figure 1 depicts the microstructures of the samples after KOBO extrusion and aging. The unreinforced alloy contains coaxial grains with an average grain size of ~10 μm, as is shown in Figure 1a. Precipitates can be seen in the image with higher magnification, and these indicate the homogeneous distribution of precipitate particles in the grains. The microstructure of the ZK60/SiC_p_ composite is depicted in Figure 1b and shows an average grain size of ~5 μm. The figure makes it clear that the extrusion process resulted in bimodal grain distributions. In some regions, partial recrystallization occurred around the ceramic particles, and the grain size was reduced significantly. Furthermore, it is evident from a comparison of Figure 1a,b that a larger degree of deformation caused a higher degree of grain recrystallization in the composite microstructure.

### 3.2. Load Transfer Factor

**Figure 2:**
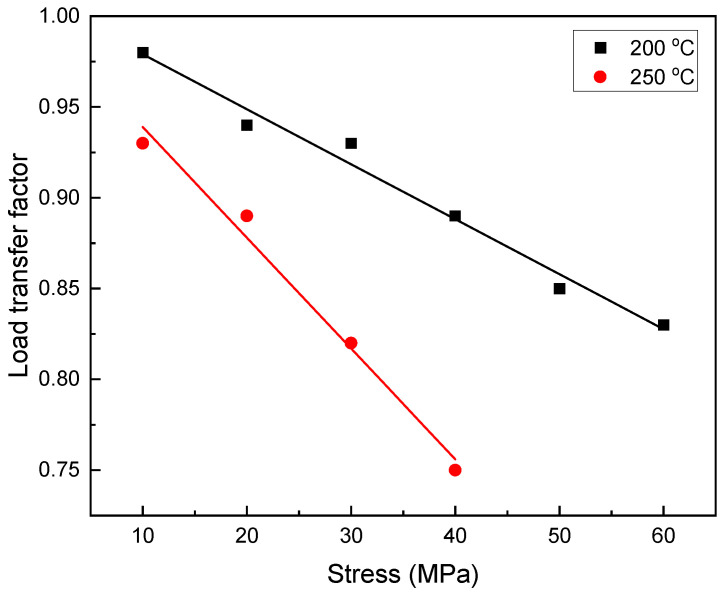
Changes in the load transfer factor in terms of applied stresses at temperatures of 200 °C and 250 °C.

The load capacity of the particles was calculated by calculating the load transfer factor using Equation (1) [27], and the changes in the load transfer factor relative to the temperature are given in Figure 2.

(1) $$\frac{{\dot{\varepsilon }}_{c}}{{\dot{\varepsilon }}_{m}}={\left(1-\alpha \right)}^{n}$$

where ${\dot{\varepsilon }}_{c}$ is the composite minimum creep rate, ${\dot{\varepsilon }}_{m}$ is the matrix minimum creep rate, α is the load transfer factor, and n is the stress exponent.

As can be seen in the figure, under low stresses, the presence of SiC particles played an important role in load transfer, which was reduced by increasing the stress of the linear load transfer factor. By reducing the load transfer factor, the matrix actually plays a role in load bearing, and this factor causes a change in the matrix [28]. It is also clear that as the temperature rises to 250 °C, the load transfer factor decreases.

### 3.3. Creep Curve

**Figure 3:**
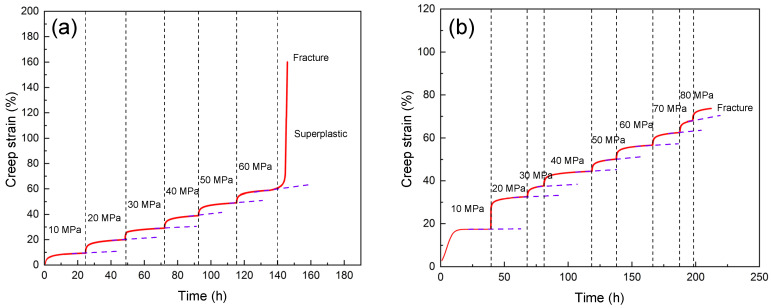
Creep curves under different stress levels at 200 °C: (**a**) unreinforced alloy and (**b**) composite sample.

Figure 3 shows the accelerated creep curves for the unreinforced alloy and the composite samples at 200 °C under different stress levels. As can be seen, in the composite creep curve, the first and third stages of creep are clearly visible, and this shows that, like the unreinforced alloy, it has three complete stages (a characteristic feature of the creep curves of non-continuous reinforcing composites) [29]. Comparing this with the unreinforced alloy creep diagram, it is clear that the slope of the third zone is less steep and that its length is shorter, and this could be related to the presence of particles and their effect on the type of failure. The wide third region of the unreinforced alloy is due to its quasi-superplastic behavior under the temperature and stress applied during the creep tests, which caused a great increase in elongation.

Table 1 shows the stress levels applied in the creep tests at 200 °C and 250 °C for the different samples. As can be seen, the different stress levels applied to the unreinforced alloy were investigated, and the alloy sample was able to withstand a stress level of 60 MPa at 200 °C. When the temperature was increased to 250 °C, the sample tolerance was reduced to 40 MPa. The amount of stress tolerated by the composite was ~28% higher than the stress tolerated by the unreinforced alloy at 200 °C and ~40% higher at 250 °C. When comparing the unreinforced alloy and composite samples at 200 °C and 250 °C, it is clear that as the temperature increased, the curve loses its stepped pattern, resulting in ~40% and ~28% increases in the amount of creep strain, respectively.

### 3.4. Stress Exponent

**Figure 4:**
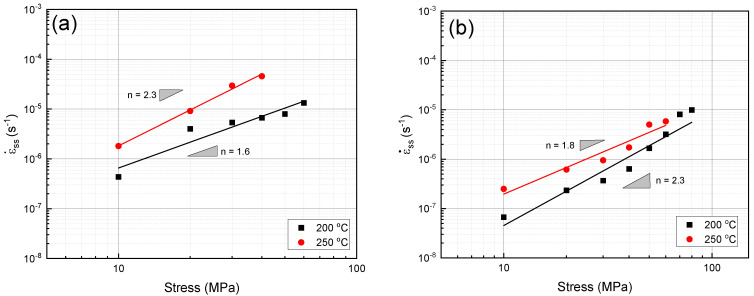
Minimum creep rate changes in terms of different stress levels in the double logarithm scale: (**a**) unreinforced alloy and (**b**) composite.

Figure 4 shows the minimum creep rate change curve in terms of the applied stress as a double logarithm scale according to Equation (2) [9] for the unreinforced alloy and the composite at temperatures of 200 °C and 250 °C.

(2) $$\dot{\varepsilon_{s}^{\;}}=A\sigma^{n}\exp(\frac{-Q}{RT})$$

where σ is the applied stress (MPa), Q is the activation energy (kJ/mol), A is constant, R is the global constant of gases (8.314 kJ/mol), n is the stress exponent, and T is the temperature (473 K and 523 K).

The calculated n values for the unreinforced alloy at temperatures of 200 °C and 250 °C were close to 2 (2.3 and 1.6, respectively, Figure 4a, which is within the scope of the GBS mechanism. As can be seen in Figure 4b, the n values of the composite sample (1.8 at 200 °C and 2.3 at 250 °C) are within the scope of the two GBS mechanisms and the viscous glide mechanism. However, due to the presence of reinforcements, the n values of the composite increase compared with those of the unreinforced alloy, likely because a similar mechanism occurs at both temperatures. Comparing the steady creep rates at 200 °C and 250 °C in Figure 4, it is clear that the creep rate increased with the increasing temperature. This is related to the fact that the diffusion coefficient of the alloy increased with the increase in temperature, which in turn caused the creep mechanisms to occur more rapidly [30].

### 3.5. Activation Energy

**Figure 5:**
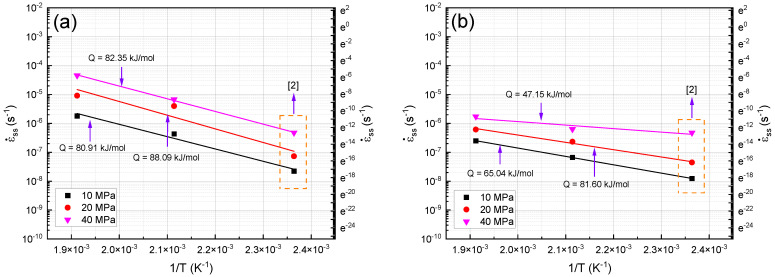
Minimum creep rate changes in terms of 1/T under different stress levels: (**a**) unreinforced alloy and (**b**) composite.

Figure 5a shows the creep activation energy values of the unreinforced alloy and the composite under different stress levels. As can be seen, the apparent creep activation energy of the unreinforced alloy under stress levels of 10 MPa, 20 MPa, and 40 MPa is in the range of 80.91–88.09 kJ/mol, which is less than that of Mg self-diffusion (135 kJ/mol) [31]. The apparent creep activation energy of the composite under stress levels of 10 MPa, 20 MPa, and 40 MPa is in the range of 47.15–65.04 kJ/mol. The calculated activation energy is significantly less than that of Mg self-diffusion (135 kJ/mol) [31], and the apparent activation energy of the composite, like that of the unreinforced alloy, is within the scope of the GBS mechanism. It is clear that the activation energy decreased significantly with the increasing stress in the composite sample. Kaibyshev and Sitdikov [30] revealed that the incidence of recrystallization and the rise in an alloy’s diffusion rate are connected to the decline in activation energy, which is lowered by the refinement of the microstructure caused by recrystallization. However, at low stress, there is an opportunity for the dislocations to climb and slip, and this climbing and slipping of the dislocations can encourage GBS and determine the type of deformation [31]. In a review study on ZK60, AZ91, and AS21 alloys, n values in the 1.5–2 range and activation energy values of 30–45 kJ/mol were reported, and these are within the scope of the GBS mechanism [3]. It is obvious that the microstructures of the samples have been refined due to the extrusion process and that the volume fraction of the grain boundaries has increased. In fact, the energy required for activating grain boundary diffusion decreased with the decreasing grain size.

### 3.6. Phenomenological Creep Equation

**Figure 6:**
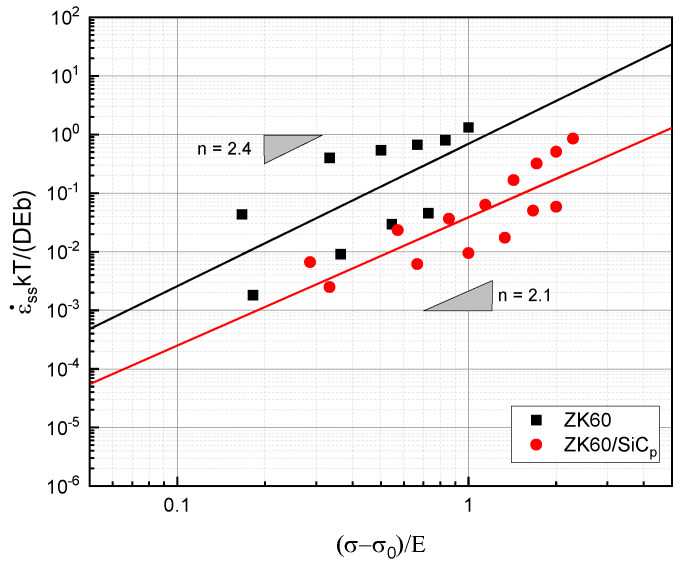
Normalized minimum creep rate with Mg self-diffusion in terms of normalized stress with elastic modulus.

In order to evaluate the alloy creep resistance independently of the test temperature, the minimum creep rate was normalized using the alloy diffusion coefficient at the test temperatures in accordance with the Dorn relation. The normalized stable creep rate data ($\dot{\varepsilon }\mathrm{kT}/\left(\mathrm{DEb}\right)$) are plotted on a double logarithmic scale in Figure 6 according to the effective normalized stress ($\left(\sigma -\sigma_{{}^{\circ}}\right)/E$). Using the obtained data and fitting a line, n values of 2.4 for the reinforced alloy and 2.1 for the composite were calculated, and these values are indicative of the GBS mechanism and conform with the Ball–Hutchinson grain boundary sliding model [32]. In fine-grained microstructures, Nabarro–Herring creep and Coble creep usually contribute to the deformation in/near the grain boundaries and are regarded as the mechanisms that stimulate GBS [31]. The unreinforced alloy’s fine-grained microstructure makes it feasible for GBS to occur at low loads via the diffusion process. GBS and grain boundary energy are closely related, i.e., a grain boundary with high energy is likely to promote GBS. To find the diffusional creep rate, it is usually assumed that grain boundary sliding occurs under any stress, and this is the reason for the stress threshold of 0 at temperatures of 200 °C and 250 °C. Because a smooth boundary acts like a liquid, the diffusional creep rate decreases as the grain boundary viscosity increases. During the plastic deformation caused by the GBS mechanism, grain rotation occurs due to the elastic heterogeneity of adjacent grains and shear stresses [33]. When comparing the creep rate of the composite to that of the unreinforced alloy, it is clear that a change in the curve occurs under greater stresses. This change suggests an increased creep resistance in the composite compared with that of the unreinforced alloy. This high creep resistance can be attributed to the load transfer from the matrix to the particle (Figure 2) and to mismatch dislocations [34]. The presence of particles in the matrix caused the grain size to become smaller, resulting in easier GBS. However, the experimental results show that the efficient load transfer from the matrix to the particle compensated for the fine-grained matrix.

### 3.7. Elongation

**Figure 7:**
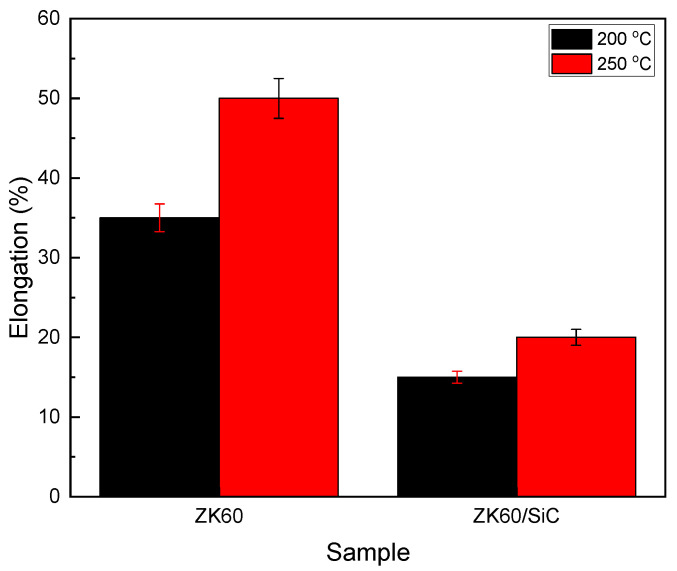
Elongation at different temperatures after 100 h creep test at a stress level of 40 MPa for the unreinforced alloy and composite samples.

**Figure 8:**
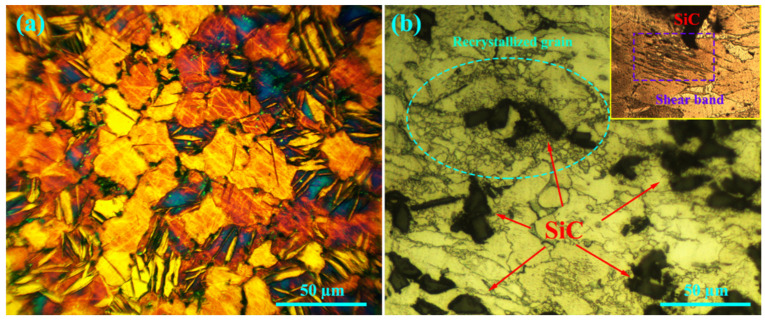
Microstructures of crept samples under 20 MPa at 200 °C after 25 h: (**a**) unreinforced alloy and (**b**) composite.

Figure 7 illustrates the changes in elongation that were observed in the samples at temperatures of 200 °C and 250 °C after the creep test and under 40 MPa of stress. According to the figure, as the temperature increased from 200 °C to 250 °C, the elongation of the unreinforced alloy increased by 10% and that of the composite increased by 20%. At both temperatures, however, the elongation percentage of the composite was lower than that of the unreinforced alloy. The increase in the resulting elongation was due to the kink band and the slip band in the microstructure (Figure 8). When comparing the creep test results of the unreinforced alloy and the composite at both 200 °C and 250 °C, it is obvious that the increased elongation was a result of the rising temperature. A comparison of the creep test results at 200 °C and 250 °C with those at 150 °C [2] shows that the elongation increased as the temperature increased. When creep deformity is caused by GBS, quasi-superplastic deformation occurs in the sample, and this indicates suitable ductility.

### 3.8. Microstructure

**Figure 9:**
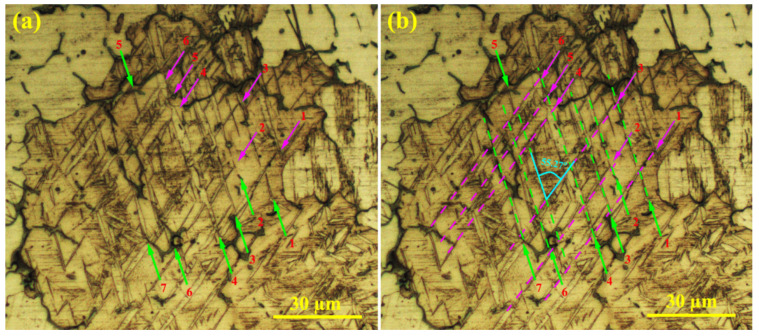
Microstructures of unreinforced alloy at high magnification: (**a**) 3D network twinning and (**b**) location of twins with lines.

Figure 8a,b show the microstructures of the unreinforced alloy and the composite at 200 °C under 20 MPa after 25 h of creep. According to the figure, high volume factions of twins and double twins formed in the microstructure. The grain size affects the activation stress of the twin formation, which is determined by the energy of the twin activation at 200 °C. The twin mechanism is active at a grain size of 10–20 µm [35]. In order to examine the 3D network of tensile and compressive twins, one of the double twin networks is given in Figure 9 with high magnification. Seven twins are named, and it can be seen that twins 1, 2, 3, 4, 5, and 6 (marked with purple arrows) each have the same orientation, as do twins 1, 2, 3, 4, 5, 6, and 7 (marked with green arrows), and that these groups of twins differ in their orientation by 60°. Assuming that the direction of the cross-section of the grain shown in the figure matches the direction perpendicular to the c-axis, there is an angle of 60° between the main axis of the twins. However, the purple arrow twins align and share the <1120> axis, and the green arrow twins are similarly aligned. In the figure, it is clear that the creation of this network of twins did not lead to the formation of cracks in the intersection and corners, and this shows that the creation of a network of twins does not cause early failure. On the one hand, the creation of a twin network in the microstructure provides the conditions for recrystallization, which increases ductility. However, on the other hand, with the refinement of the microstructure and the influence of the Hall–Petch relation [36], the strength of the sample increases.

A closer look at the composite microstructure is provided in Figure 8b by the box in the top right corner, which shows the shear bands. These areas are actually localized flows caused by severe plastic deformation. Shear bands can be inserted into grains, and their orientation is independent of the orientation of the grain [37,38,39]. When local stress is high enough to exceed the activation energy required to create a secondary twin, a change in the c direction occurs. The twins are oriented towards the slip direction of the base planes, and this leads to a pile-up of dislocations at the twin boundary. This accumulation causes a high concentration of stress, which stimulates the formation of shear bands via homogeneous distribution in the microstructure [38].

**Figure 10:**
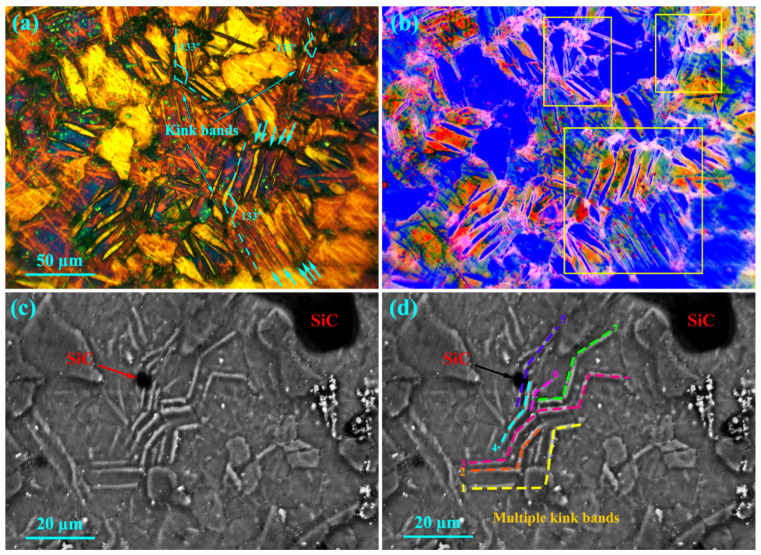
Microstructures of crept samples under 40 MPa at 200 °C after 25 h: (**a**,**b**) unreinforced alloy and (**c**,**d**) composite.

Figure 10a,b show the crept microstructure of the unreinforced alloy at 200 °C under 40 MPa. It is clear that by increasing the stress, multiple kink bands are created in the composite microstructure. The creation of a kink band causes the refinement of the microstructure, and this increases its strength, according to the Hall–Petch relation [40,41]. According to Figure 10a,b, a kink band with an angle of 133° formed in the unreinforced alloy. No crack formed around it because the creation of a relatively homogeneous plastic deformation in the microstructure caused the release of localized strain in the surrounding area. Figure 10c,d show that multiple kink bands formed in the composite microstructure. During the extrusion, the particles were aligned due to stress, but this alignment of the particles during the extrusion process was not ideal, and to some extent the particles may have deviated from the load direction during the creep loading. This angle deviation in the direction of the load applied during the creep test caused the local yield to occur in these areas, making them very prone to kink band formation. In composites, the application of loads in the direction of the particles causes non-uniform strain distribution. This means that the potential for deformations to occur in the areas around the particles is much lower due to the high strength of the matrix, but in the areas farther from the particles there is a higher potential for deformations to occur due to the softness of the matrix. When localized yield occurs, the particle is more capable than the matrix to withstand the load. This factor has caused the matrix to increase the elongation. However, ceramic particles do not elongate significantly. As a result, the conditions for the formation of multiple kink bands are created in the microstructure [42]. By creating a kink band, the localized stress is released, reducing the internal energy to a minimum [43]. As the deformation continues, the dislocations in the kink band begin to arrange themselves, causing sub-boundaries to form, and the sub-grains begin to rotate to accommodate the microstructure, leading to the formation of new, smaller grains [44]. In addition to its increasing strength, this microstructure also exhibits improved ductility. It can be concluded from the results of the creep test at 200 °C that the creation of a kink band simultaneously increases the strength and ductility of a composite.

**Figure 11:**
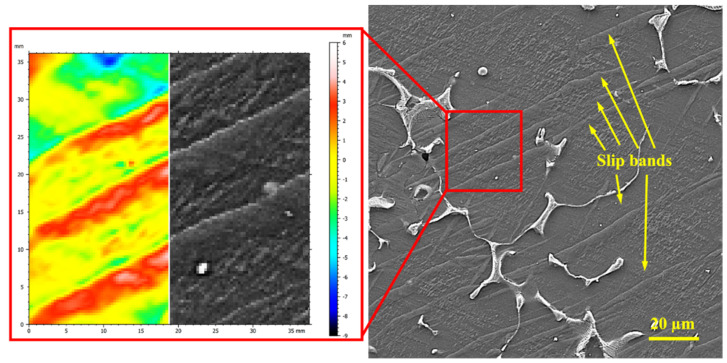
SEM micrographs of crept microstructure of unreinforced alloy under 40 MPa at 250 °C after 25 h and analysis profile of the slip lines in the grain (created using SPIP software).

Figure 11 shows the microstructure of the unreinforced alloy at 250 °C under 40 MPa after 25 h of creep. In the figure, slip bands are seen, along with grain boundary serration and nodules, due to the presence of particles in the unreinforced alloy. The inconsistencies with the HCP structure, caused by the inherent heterogeneity of the magnesium, have caused more stress concentrations at the grain boundaries between the nodules, and this is the cause of the GBS. The stress concentration caused by the interaction of the slip band and the grain boundary causes GBS to occur, and the stress concentration subsequently decreases. Various methods have been used in different studies to activate GBS, one of which is to characterize slip bands within the grain [45], and an example of this can be seen in Figure 11. At the intersection of the precipitates, the high-angle grain boundary of the grain in which the dislocation density is greater progresses to the lateral grain due to the recovery process, creating a grain boundary migration [46,47]. In this mechanism, prismatic slip accompanies GBS. At this temperature, the activation energy of the slip on the pyramidal planes is high, so the prismatic slip activity is predominant [48]. Previous studies have also reported GBS in the matrix/participation interface [49].

To confirm this mechanism, the slip bands in Figure 11 were analyzed using SPIP software, and the results are shown in the red box. Figure 11 shows an SEM micrograph of the sliding lines, indicating the severe effects of slipping on the precipitate nodules. When analyzing the slip lines, it is clear that there is a difference in height between the two marked parts, and this difference in height can be related to the creation of a slip band. In the areas around the precipitate, it is clear that the height difference continues to the grain boundary, indicating the formation of grain boundary sliding. Studies have shown that the observed height difference is due to cross-slip that results from the pile-up of dislocations [45]. In fact, the dislocation pile-ups are caused by the high density of the dislocations on the basal planes [45,50]. The nodules that are created in the direction of the tensile axis do not deform easily in the basal and prismatic planes. As a result, shear stress is stored in the nodule area and activates the <c + a> pyramidal slip system [50]. Consequently, the crystallization of the nodules in a limited range causes a GBS that is related to the angle between the c-axis and the loading direction during the creep (45–90°) [51,52,53]. At 200 °C, the deformation is controlled by the cross-slip of the dislocations. The cross-slip of dislocations via the Friedel–Escaig mechanism leads to a dislocation of the orientation of the basal planes. The continued absorption of dislocations at the slip band boundary causes recrystallization in the microstructure. The refinement created by the recrystallization provides the conditions for GBS [54].

### 3.9. Failure Mechanism

**Figure 12:**
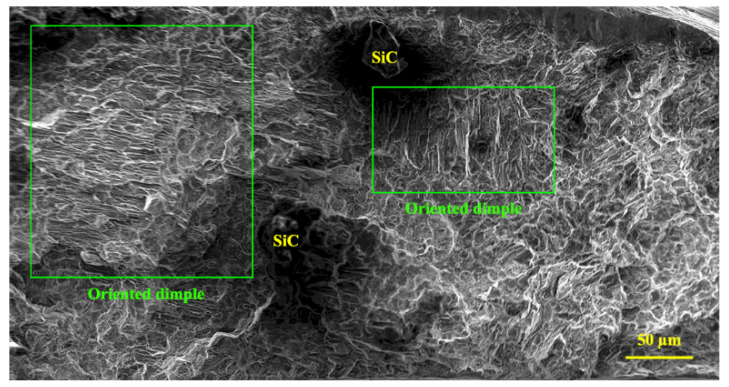
Fracture surface of composite sample after creep at 200 °C under 40 MPa.

Figure 12 shows the fracture surfaces of the composite sample after the creep test under 40 MPa at 200 °C. Two features can be seen by studying the fracture surface of the composite sample. First, the large concave area contains smaller creep cavities which have formed around the ceramic particles. The large convex area is farther from the ceramic particles, which shows the strong interface of the particles with the matrix and the quasi-superplastic behavior of the matrix. A strong interface between the particles and the matrix was evident since there was no apparent sign of debonding in the fracture surface. The presence of micro-cracks around the particles may be due to the stress concentration at the edges of the particles. The formation of micro-cracks has led to the pile-up of dislocations on the plane near the Lamela boundary. Due to the density of the dislocations, the Lamela boundaries have caused localized deformations, and the stress concentration has caused the cracks to nucleate. The stress is transferred to the particles through the matrix, and the difference between the elastic modulus of the matrix and that of the particles causes the cracks to grow. The pile-up of dislocations in the matrix/reinforcement interface causes crack nucleation in the particle–matrix interface [55,56].

Elongated dimples are related to the direction of the applied stress. Cavitation during creep is the cause of the extensive dispersion of fine dimples and indicates the level of intergranular fracture [57]. Very fine dimples are related to the presence of reinforcements in the matrix. The ceramic particles have borne the load transferred through the matrix, have not allowed the growth of cavities, and have increased the cavity nucleation rate. Fracturing has occurred through localized plastic behavior at the fracture surface. As the deformation continues at high temperatures, the micro-cavities do not coalesce quickly, but instead micro-necking occurs, creating particles that expand and fracture. These particles are the steps between the micro-cavities. Furthermore, the composite yield stress decreases as the temperature increases, and the composite becomes more elastic under constant stress, allowing cavities to grow. As the creep test time increases, crack growth is achieved via the coalescence of micro-cavities, and this ultimately leads to microscopic failure. However, due to the presence of the particles, the growth of the cavities in the composite is limited, leading to brittle failure in some areas. In the figure, broken particles surrounded by small cavities can be seen.

**Figure 13:**
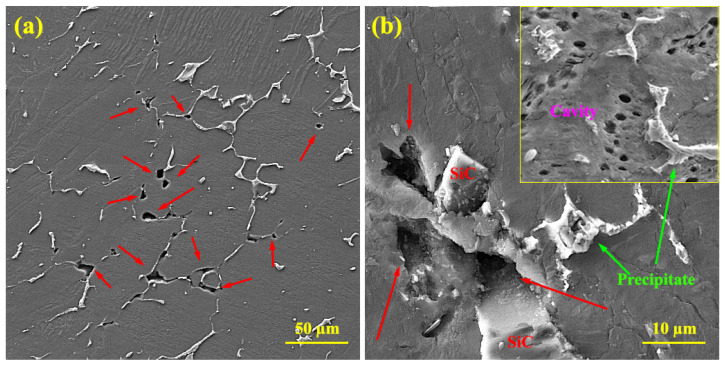
SEM micrographs of cavitation after creep under 40 MPa at 250 °C: (**a**) unreinforced alloy and (**b**) composite. Red arrows indicate cavities and cracks.

Figure 13 shows the areas 5 mm below the fracture surfaces of the unreinforced alloy and the composite. The figure shows that the micro-cavity nucleation mechanisms in the unreinforced alloy and the composite are different. In the unreinforced alloy, cavities are generally found at the intersection of precipitates, grain boundaries, triple boundaries, and especially at grain boundaries perpendicular to the loading direction. Thus, it is clear that the stress concentration created by the intersection of the slip bands at the grain boundary has led to the formation of intergranular micro-cracks. The micro-cavity caused by precipitation nucleation has not grown much after the deformation. The coalescence of micro-cavities in precipitations in different sections of the sample can play a vital role in the initiation of microscopic failure. Micro-cracks grew earlier in the unreinforced alloy than in the composite, so dynamic strain aging (DSA) activation could be the cause of this rapid growth [58]. In fact, the pile-up of dislocations in the microstructure has led to a high stress concentration in the grain boundaries, which is itself a factor in crack nucleation. In the composite, micro-cavities are seen in the grain, as is shown in Figure 13b. In the composite sample, the nucleation of cavities in the grain is caused by the load bearing of reinforcing particles at low stresses, and as the stress increases (see Figure 2), the amount of load transfer decreases at once, causing the whole matrix to be exposed to high stress and become susceptible to cavity nucleation. An examination of the composite microstructure presented in Figure 13b shows that the areas around the particles are prone to crack nucleation. The areas around the reinforcing particles are prone to stress concentration, and are thus areas susceptible to cavitation.

## 4. Conclusions

In this study, we examined the creep behaviors and microstructural changes in unreinforced ZK60 alloy and a ZK60/SiC_p_ composite at 200 °C and 250 °C after KOBO extrusion and precipitation hardening. The results can be summarized as follows:

1. The load transfer factor was approximately 0.97 at 200 °C and 0.93 at 250 °C under low stresses, and this decreased linearly as the stress increased. This decrease was caused by a decrease in the load ability of the reinforcement and an increase in the load imposed on the matrix with the increase in the applied load.
2. The true stress exponent for both the unreinforced alloy and the composite at 200 °C and 250 °C was in the range of 2.1–2.4, and the activation energy was in the range of 80.91–88.09 kJ/mol for the unreinforced alloy and 47.15–81.60 kJ/mol for the composite, indicating the GBS mechanism.
3. At 200 °C, the strengthening mechanisms under low stresses were the twinning, double twinning, and shear bands, and as the stress increased, mechanisms such as slip bands and kink bands became active. At 250 °C, it was found that the slip band created during the creep deformation effectively delayed grain boundary sliding.
4. A study of the fracture surfaces and microstructures of areas near the fracture surfaces at different temperatures showed that cavitation was the main failure mechanism. The cause of cavitation in the unreinforced alloy differed from that of the composite, and this can be attributed to the presence of particles in the composite. Major cavities were detected at the precipitate intersection and the matrix/particle interface due to the localized stress concentration created in these areas.
5. The elongation of the samples after a 100 h creep test showed that at 200 °C the composite elongated more than 15% and the unreinforced alloy elongated more than 35%, and at 250 °C the composite elongated more than 20% and the unreinforced alloy elongated more than 50%.

## Figures and Tables

**Table 1:** Stress levels applied to samples in accelerated creep test until fracture.

Sample	Temperature (°C)	Stress (MPa)							
ZK60 alloy	200	10	20	30	40	50	60	–	–
	250	10	20	30	40	–	–	–	–
ZK60/SiC_p_ composite	200	10	20	30	40	50	60	70	80
	250	10	20	30	40	50	60	–	–

## Data Availability

Not applicable.

## References

[1] Guan D., Liu X., Gao J., Ma L., Wynne B.P., Rainforth W.M. (2019). Exploring the mechanism of “Rare Earth” texture evolution in a lean Mg–Zn–Ca alloy. Sci. Rep..

[2] Wang Y.-Y., Jia C., Tayebi M., Hamawandi B. (2022). Microstructural Evolution during Accelerated Tensile Creep Test of ZK60/SiCp Composite after KoBo Extrusion. Materials.

[3] Mukai T., Nieh T.G., Iwasaki H., Higashi K. (1998). Superplasticity in doubly extruded magnesium composite ZK60/SiC/17p. Mater. Sci. Technol..

[4] Zhao D., Jiang C., Zhao K. (2023). Ultrasonic welding of AZ31B magnesium alloy and pure copper: Microstructure, mechanical properties and finite element analysis. J. Mater. Res. Technol..

[5] Liu S., Wang Y., Yarigarravesh M., Tayyebi M., Tayebi M. (2022). Evaluation of whisker alignment and anisotropic mechanical properties of ZK60 alloy reinforced with SiCw during KOBO extrusion method. J. Manuf. Process..

[6] Wang Y., Tayyebi M., Tayebi M., Yarigarravesh M., Liu S., Zhang H. (2023). Effect of whisker alignment on microstructure, mechanical and thermal properties of Mg-SiCw/Cu composite fabricated by a combination of casting and severe plastic deformation (SPD). J. Magnes. Alloy..

[7] Xu Y., Liang Y., Peng G. (2020). Effect of a compound modification process on the microstructure and mechanical properties of ZK60 magnesium alloys. Mater. Sci. Eng. A.

[8] Tekumalla S., Gupta N., Gupta M. (2020). Influence of turning speed on the microstructure and properties of magnesium ZK60 alloy pre-processed via turning-induced-deformation. J. Alloys Compd..

[9] Tayebi M., Najafi H., Nategh S., Khodabandeh A. (2021). Creep Behavior of ZK60 Alloy and ZK60/SiCw Composite After Extrusion and Precipitation Hardening. Met. Mater. Int..

[10] Koike J., Ohyama R., Kobayashi T., Suzuki M., Maruyama K. (2003). Grain-Boundary Sliding in AZ31 Magnesium Alloys at Room Temperature to 523 K. Mater. Trans..

[11] Bussiba A., Ben Artzy A., Shtechman A., Ifergan S., Kupiec M. (2001). Grain refinement of AZ31 and ZK60 Mg alloys—towards superplasticity studies. Mater. Sci. Eng. A.

[12] Chino Y., Iwasaki H., Mabuchi M. (2004). Cavity growth rate in superplastic 5083 Al and AZ31 Mg alloys. J. Mater. Res..

[13] Srinivasan A., Swaminathan J., Pillai U.T.S., Guguloth K., Pai B.C. (2008). Effect of combined addition of Si and Sb on the microstructure and creep properties of AZ91 magnesium alloy. Mater. Sci. Eng. A.

[14] Watanabe H., Moriwaki K., Mukai T., Ohsuna T., Hiraga K., Higashi K. (2003). Materials Processing for Structural Stability in a ZK60 Magnesium Alloy. Mater. Trans..

[15] Liu X., Wang J. (2016). Low-energy, Mobile Grain Boundaries in Magnesium. Sci. Rep..

[16] Torbati-Sarraf H., Torbati-Sarraf S.A., Poursaee A., Langdon T.G. (2019). Electrochemical behavior of a magnesium ZK60 alloy processed by high-pressure torsion. Corros. Sci..

[17] Tayebi M., Nategh S., Najafi H., Khodabandeh A. (2020). Tensile properties and microstructure of ZK60/SiCw composite after extrusion and aging. J. Alloys Compd..

[18] Chmelík F., Lukáč P., Janeček M., Moll F., Mordike B.L., Kainer K.-U., Langdon T.G. (2002). An evaluation of the creep characteristics of an AZ91 magnesium alloy composite using acoustic emission. Mater. Sci. Eng. A.

[19] Shamekh M., Pugh M., Medraj M. (2013). Processing and Characterization of In Situ (TiC–TiB2)p/AZ91D Magnesium Matrix Composites. Adv. Eng. Mater..

[20] Dieringa H., Huang Y., Maier P., Hort N., Kainer K.U. (2005). Tensile and compressive creep behaviour of Al2O3 (Saffil^®^) short fiber reinforced magnesium alloy AE42. Mater. Sci. Eng. A.

[21] Sato T. (2008). Power-Law Creep Behaviour in Magnesium and Its Alloys. Ph.D. Thesis.

[22] Kim J., Kaneko J., Sugamata M. (1992). High Temperature Deformation of SiC Whisker/ AZ91 Magnesium Alloy and SiC Whisker/2324 Aluminum Alloy Composites. J. Jpn. Inst. Met..

[23] Nieh T.G., Schwartz A.J., Wadsworth J. (1996). Superplasticity in a 17 vol.% SiC particulate-reinforced ZK60A magnesium composite (ZK60/SiC/17p). Mater. Sci. Eng. A.

[24] Moheimani S.K., Azadeh K., Khademzadeh S., Tayebi M., Rajaee A., Saboori A. (2022). Tribological behaviour of AZ31 magnesium alloy reinforced by bimodal size B4C after precipitation hardening. J. Magnes. Alloy..

[25] Korbel A., Bochniak W., Ostachowski P., Błaż L. (2011). Visco-Plastic Flow of Metal in Dynamic Conditions of Complex Strain Scheme. Met. Mater. Trans. A.

[26] Wang X., Yang J., Chi P., Bahonar E., Tayebi M. (2021). Effects of the microstructure and precipitation hardening on the thermal expansion behavior of ZK60 magnesium alloy. J. Alloys Compd..

[27] TIAN J., SHI Z. (2014). Creep mechanism and creep constitutive model of aluminum silicate short-fiber-reinforced magnesium matrix composite. Trans. Nonferrous Met. Soc. China.

[28] Al-Samman T., Molodov K.D., Molodov D.A., Gottstein G., Suwas S. (2012). Softening and dynamic recrystallization in magnesium single crystals during c-axis compression. Acta Mater..

[29] Chawla N., Chawla K.K. (2006). Metal Matrix Composites.

[30] Kaibyshev R., Sitdikov O. (1994). Dynamic Recrystallization of Magnesium at Ambient Temperature. Z. Met..

[31] Athul K.R., Pillai U.T.S., Srinivasan A., Pai B.C. (2016). A Review of Different Creep Mechanisms in Mg Alloys Based on Stress Exponent and Activation Energy. Adv. Eng. Mater..

[32] Mukherjee A.K. (1993). Superplasticity in Metals, Ceramics and Intermetallics.

[33] Masuda H., Tobe H., Sato E., Sugino Y., Ukai S. (2019). Diffusional mass flux accommodating two-dimensional grain boundary sliding in ODS ferritic steel. Acta Mater..

[34] Khorshidi R., Mahmudi R., Honarbakhsh-Raouf A. (2016). Compressive creep behavior of a cast Al–15Mg2Si in situ composite. Mater. Sci. Eng. A.

[35] Barnett M.R. (2003). A taylor model based description of the proof stress of magnesium AZ31 during hot working. Met. Mater. Trans. A.

[36] Chen Y., Sun S., Zhang T., Zhou X., Li S. (2020). Effects of post-weld heat treatment on the microstructure and mechanical properties of laser-welded NiTi/304SS joint with Ni filler. Mater. Sci. Eng. A.

[37] Semiatin S.L., Jonas J.J. (1984). Formability and Workability of Metals: Plastic Instability and Flow Localization.

[38] Sandlöbes S., Zaefferer S., Schestakow I., Yi S., Gonzalez-Martinez R. (2011). On the role of non-basal deformation mechanisms for the ductility of Mg and Mg–Y alloys. Acta Mater..

[39] Su J., Sanjari M., Kabir A.S.H., Jung I.-H., Yue S. (2016). Dynamic recrystallization mechanisms during high speed rolling of Mg–3Al–1Zn alloy sheets. Scr. Mater..

[40] Yu H., Xin Y., Wang M., Liu Q. (2018). Hall-Petch relationship in Mg alloys: A review. J. Mater. Sci. Technol..

[41] Shi B.Q., Cheng Y.Q., Shang X.L., Yan H., Chen R.S., Ke W. (2019). Hall-Petch relationship, twinning responses and their dependences on grain size in the rolled Mg-Zn and Mg-Y alloys. Mater. Sci. Eng. A.

[42] Takahashi T., Ueda M., Iizuka K., Yoshimura A., Yokozeki T. (2019). Simulation on kink-band formation during axial compression of a unidirectional carbon fiber-reinforced plastic constructed by X-ray computed tomography images. Adv. Compos. Mater..

[43] Ueta R., Shizawa K. (2016). Investigation on Position of Kink Band Formation in Single Crystal of Mg-Based LPSO Phase Using Dislocation-Based Crystal Plasticity Simulation. J. Soc. Mater. Sci. Jpn..

[44] Wu S., Zhang Z., Zhang J., Xu C., Niu X., Liu W. (2017). Effects of Phase Content and Evolution on the Mechanical Properties of Mg 95 Y 2.5 Zn 2.5 and Mg 93.1 Y 2.5 Zn 2.5 Ti 1.6 Zr 0.3 Alloys Containing LPSO and W Phases: Effects of Phase Content and Evolution. Adv. Eng. Mater..

[45] Hémery S., Tromas C., Villechaise P. (2018). Slip-stimulated grain boundary sliding in Ti-6Al-4V at room temperature. Materialia.

[46] He T., Feng M. (2018). Combined effects of cooperative grain boundary sliding and migration and reinforced particles on crack growth in fine-grained Mg alloys. J. Alloys Compd..

[47] Zhang B., Wang Z., Yu H., Ning Y. (2022). Microstructural origin and control mechanism of the mixed grain structure in Ni-based superalloys. J. Alloys Compd..

[48] Barnett M.R., Bettles C., Barnett M.B.T.-A. (2012). 3-Twinning and its role in wrought magnesium alloys. Woodhead Publishing Series in Metals and Surface Engineering.

[49] Wu X.J., Koul A.K. (1995). Grain boundary sliding in the presence of grain boundary precipitates during transient creep. Met. Mater. Trans. A.

[50] Castany P., Pettinari-Sturmel F., Douin J., Coujou A. (2017). TEM quantitative characterization of short-range order and its effects on the deformation micromechanims in a Ti-6Al-4V alloy. Mater. Sci. Eng. A.

[51] Echlin M.P., Stinville J.C., Miller V.M., Lenthe W.C., Pollock T.M. (2016). Incipient slip and long range plastic strain localization in microtextured Ti-6Al-4V titanium. Acta Mater..

[52] Hémery S., Dang V.T., Signor L., Villechaise P. (2018). Influence of Microtexture on Early Plastic Slip Activity in Ti-6Al-4V Polycrystals. Met. Mater. Trans. A.

[53] Li H., Boehlert C.J., Bieler T.R., Crimp M.A. (2015). Examination of the distribution of the tensile deformation systems in tension and tension-creep of Ti-6Al-4V (wt.%) at 296 K and 728 K. Philos. Mag..

[54] Püschl W., Schoeck G., Kirchner H.O.K. (1987). The line tension of dislocations in anisotropic media. Philos. Mag. A.

[55] Wang J., Guo X., Qin J., Zhang D., Lu W. (2015). Microstructure and mechanical properties of investment casted titanium matrix composites with B4C additions. Mater. Sci. Eng. A.

[56] Liang L., Xu M., Chen Y., Zhang T., Tong W., Liu H., Wang H., Li H. (2021). Effect of welding thermal treatment on the microstructure and mechanical properties of nickel-based superalloy fabricated by selective laser melting. Mater. Sci. Eng. A.

[57] Das A., Kumar Chakravartty J. (2017). Fractographic correlations with mechanical properties in ferritic martensitic steels. Surf. Topogr. Metrol. Prop..

[58] Koyama M., Akiyama E., Tsuzaki K. (2014). Effects of Static and Dynamic Strain Aging on Hydrogen Embrittlement in TWIP Steels Containing Al.

